# Cobalt deficiency in ruminants from Tocantins State, Brazil: an underdiagnosed nutritional challenge?

**DOI:** 10.1007/s11250-026-05006-9

**Published:** 2026-03-31

**Authors:** Jade de Menezes Paes Bastos, Marcos Antônio Aguiar Júnior, Marina Galindo Chenard, Luiz Filipe Cabral de Souza Ramos, Kicia Russano, Adriano Tony Ramos, Nayro Xavier de Alencar, Renato das Chagas Silva, Daniel Augusto Barroso Lessa, Michel Abdalla Helayel

**Affiliations:** 1 Federal Fluminense University (UFF), Niterói, RJ Brazil; 2 Federal University of Santa Catarina (UFSC), Florianopolis, SC Brazil; 3 Self-Employed Veterinarian, Rio de Janeiro, Brazil; 4 Faculdade de Veterinária, UFF. Ave. Almirante Ary Parreiras, 50, Icaraí, Niterói, RJ 24220-000 Brazil

**Keywords:** Vitamin B12, Cachexia, Mineral deficiency, Sheep, Cattle

## Abstract

**Context:**

Although mineral deficiencies are well-documented in Brazil, they continue to cause significant economic losses, particularly in animals raised under extensive grazing systems. Cobalt (Co) acts as a cofactor for the enzyme methylmalonyl-CoA isomerase, the sole metabolic pathway for propionate conversion and subsequent glucose synthesis. It is also essential for vitamin B12 synthesis, which supports hemoglobin formation, carbohydrate and lipid metabolism, and nucleic acid synthesis. Deficient animals exhibit anorexia, weight loss (despite forage availability), anemia, ruminal atony, and lignophagia.

**Aims:**

To describe the first reported cases of cobalt deficiency in sheep and cattle in Tocantins State, Brazil, focusing on clinical history, nutritional and hematological aspects, and impacts on animal health.

**Methods:**

The study was conducted across three distinct farms in Tocantins, Northern Brazil. Diagnosis involved clinical history, physical examination, hematological analysis, blood glucose and ketone measurements, urinalysis, rumen fluid evaluation, fecal parasitology, necropsy, and liver cobalt quantification via spectrophotometry. Therapeutic diagnosis was confirmed through vitamin B12 administration and cobalt supplementation.

**Main results:**

All three farms exhibited cobalt deficiency cases, with primary symptoms including lignophagia, anorexia, progressive emaciation, and mortality. Hematological findings revealed normocytic normochromic to macrocytic hypochromic anemia, occasional leukocytosis, hypoglycemia, and normal β-hydroxybutyrate (BHB) levels. Urinalysis showed no abnormalities. Rumen fluid analysis indicated complete microbial inactivity with indigestion. All treated animals achieved full clinical recovery without relapse.

**Conclusions:**

Cobalt deficiency in sheep and cattle in Tocantins poses a significant health and productivity threat. The positive response to vitamin B12 and cobalt therapy underscores the importance of proper nutritional management.

**Implications:**

This first report of cobalt deficiency in previously unmapped regions alerts farmers and veterinarians to prioritize mineral monitoring and supplementation to mitigate economic losses and ensure animal welfare.

## Introduction

Brazil hosts the world’s largest cattle herd, producing over 2.38 million metric tons of beef carcasses and 6.19 billion liters of milk in the first quarter of 2024 alone (IBGE 2024). The country also holds significant potential for sheep farming due to favorable environmental conditions and vast land availability, which reduce production costs. However, mineral deficiencies—though well-documented in Brazil—remain a major economic burden, particularly in extensively grazed livestock. This is largely attributed to the predominance of Brazilian pastures composed of grasses with inadequate mineral profiles (Tokarnia et al. 2010).

Cobalt (Co), a trace mineral critical for ruminants, it is essential for vitamin B12 production, which supports hemoglobin synthesis, carbohydrate and lipid metabolism, and nucleic acid function (Suttle 2010). There are two enzymes dependent on this vitamin, methylmalonyl-CoA isomerase, the sole enzyme enabling propionate metabolism and subsequent glucose synthesis, and methionine synthase, which catalyzes the transfer of methyl groups from 5-methyltetrahydrofolate to homocysteine, to form methionine and tetrahydrofolate (Silva et al. 2020).

Cobalt-deficient animals exhibit anorexia and weight loss despite forage availability, anemia (typically macrocytic normochromic with hypersegmented neutrophils), ruminal atony, and lignophagia (Patterson 2017; Ventura et al. 2021). Diagnosis relies on herd observation, clinical history, and compatible pathological findings (Tokarnia et al. 2010; Suttle 2010), with confirmation via hepatic cobalt quantification, serum vitamin B12 assays, or therapeutic response to cobalt supplementation (Radostits 2007; Suttle 2010; Tokarnia et al. 2010).

Despite reports of cobalt deficiency in other Brazilian regions (Pinheiro et al. 2011; Silva et al. 2020), the condition has never been described in Tocantins State—a major cattle-producing and exporting region (Araujo et al. 2023; MAPA, 2024). This study aims to provide the first documentation of cobalt deficiency in sheep and cattle from Tocantins, detailing clinical history, nutritional and hematological profiles.

## Materials and methods

The study was conducted across three farms in Tocantins State, Northern Brazil. Farm 1: Located in Araguaína (7°11’31"S, 48°12’28"W), raising Santa Inês sheep in a semi-intensive system for meat, breeding, and elite stock. Farm 2: Situated in Ananás (6°22’7"S, 48°4’11"W), managing 5,000 Nelore cattle in an extensive beef production system. Farm 3: A family-owned farm in Babaçulândia (7°12’27"S, 47°45’49"W), maintaining 38 cattle under extensive grazing.

Clinical evaluation included history, anamnesis, and physical exams according to Feitosa (2008). For urinalysis, spontaneously voided urine samples were collected as per Kaneko et al. (2008). Blood analysis was performed by jugular venipuncture (25 × 8 mm needle, 10 mL syringe) for collection of 5 mL of blood. One drop was used for portable glucose (Chenard et al. 2024) and β-hydroxybutyrate (BHB) assays (González et al. 2000). Remaining blood was EDTA-preserved for hematology (Kaneko et al. 2008), repeated post-treatment in three animals. The number of animals in the herd, affected and evaluated animals, samples collected, necropsies performed and animals treated are described in Table 1.

**Table 1:** Number of animals in the herd, affected and evaluated animals, samples collected, necropsies performed, and animals treated on three farms in Tocantins with suspected cobalt deficiency in the herd

	Herd	Affected animals	Evaluated animals	Collected samples (*N*)	Necropsies	Treated animals*
Case I	120	70	5	Urine (5), blood (5), ruminal fluid (2) and feces (5)	0	60
Case II	5000	3000	5	Blood (5)	1	2500
Case III	38	10	10	Blood (3)	0	10

N – Number of samples collected. * All treated animals responded well to treatment with complete clinical recovery

Ruminal fluid collection was performed using an esophageal probe adapted to a suction pump and color, odor, consistency, pH, sedimentation/flotation time, methylene blue reduction (Dirksen et al. 1993) and protozoan activity (percentage alive, density, motility) were analyzed. Fecal parasitology exam was made according to Gordon and Whitlock (1939) technique. Necropsy was conducted in one animal following Barros (1988) method, with a liver sample collected for cobalt spectrophotometry following Minervino et al. (2009).

For therapeutic diagnosis following Tokarnia et al. (2010), the protocol included intramuscular (IM) administration of 5 mg/kg vitamin B12 once daily (SID) for 3 days, supplemented with 30 g cobalt sulfate per 100 kg of mineral salt.

For statistical analysis of the data, the values ​​obtained were tabulated categorically in spreadsheets. Descriptive parametric statistics were performed with determination of mean, standard deviation, minimum, maximum and median.

## Results

Three properties reported cases of cobalt deficiency. In Case I, a herd of 120 animals was evaluated, with approximately 70 (58,33%) falling ill and 10 (8,33%) succumbing to the condition. The soil was sandy, with flat pastures dominated by *Urochloa decumbens* and *Cynodon dactylon*, interspersed with invasive weeds. The sheep were provided with species-specific feed starting at three months of age, administered at 1% of their body weight, alongside ad libitum access to pasture and water troughs. A mineral supplement formulated for sheep was used; however, it was diluted 1:1 with white salt (NaCl) and offered in uncovered troughs.

Affected animals, aged 10–16 months (both sexes), exhibited hyporexia, lignophagia, lethargy, scant feces, and weight loss. The condition recurred annually, predominantly during the dry season. Some animals experienced transient improvement followed by relapse, while others progressively emaciated, culminating in death within 30–60 days. Treatments attempted by the owner, including saline purgatives, Mercepton, antibiotics, and anti-inflammatories, yielded no clinical improvement.

**Fig. 1:**
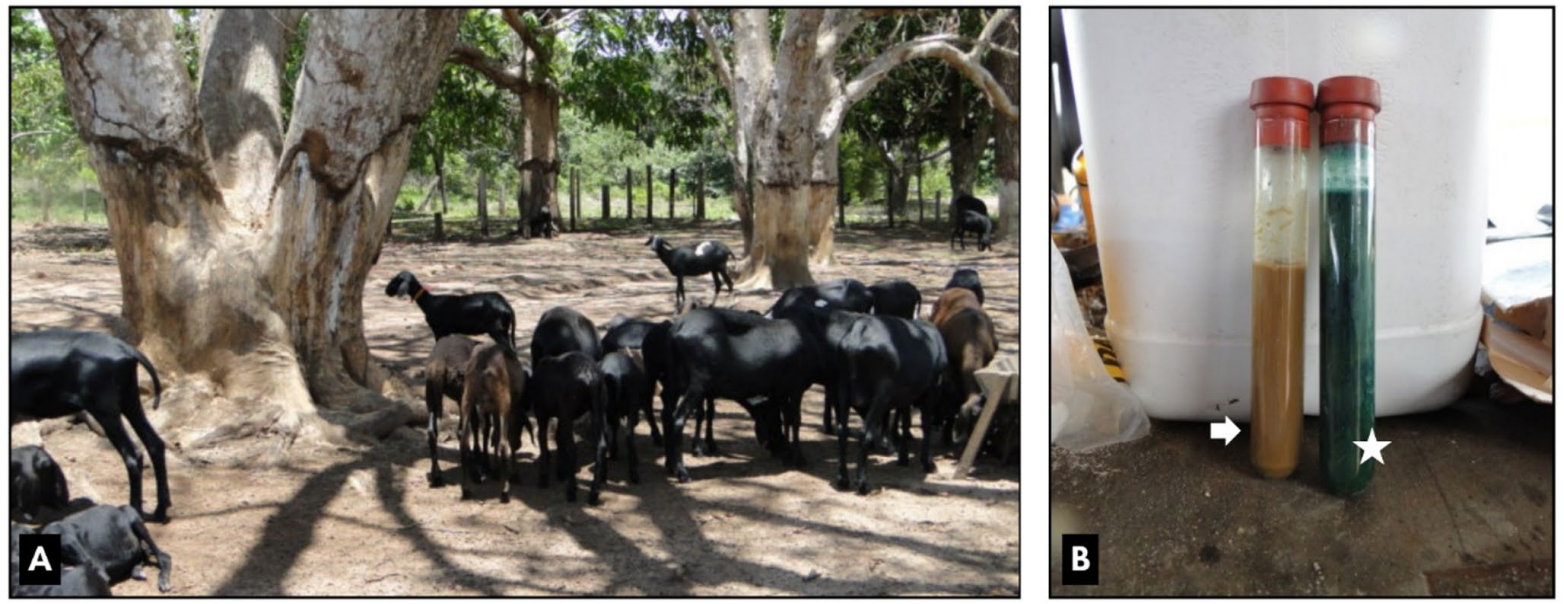
Clinical and ruminal fluid findings in cobalt-deficient sheep. (**A**) Tree trunks exhibiting marked lignophagia (wood chewing behavior) by affected sheep. (**B**) Greenish-brown ruminal fluid sample (arrow) with negative methylene blue reduction test (MBRT) result (star), indicating microbial inactivity. Source: Author’s archives, 2024

Clinical examination revealed behavioral shifts from normal to depressed (5/5; 100%), lignophagia (4/5; 80%) (tree bark and wood chewing; Fig. 1A), pale mucous membranes (5/5; 100%), capillary refill time (CRT) of 3 s (4/5; 80%), tachycardia (120 bpm - mean) (3/5; 60%), tachypnea (66 movements per minute - mean) with hyperpnea and harsh lung sounds (3/5; 60%), and ruminal atony without stratification (4/5; 80%). Remaining physical parameters were within normal limits for the species.

Urinalysis showed no abnormalities. Hematological analysis indicated normocytic normochromic anemia. Rumen fluid was greenish-brown, odorless, and watery, with a pH of 7.2, negative methylene blue reduction test (MBRT; Fig. 1B), rapid sedimentation, absent flotation, and reduced density, size, and activity of protozoa. Fecal examination revealed mild parasitic infection (< 500 ovos/g). Hypoglycemia (35 mg/dL) was observed, while beta-hydroxybutyrate (BHB) levels remained normal (0.4 mmol/L).

Clinical suspicion of cobalt deficiency prompted therapeutic diagnosis, involving the administration of 0.2 mg/animal of vitamin B12 intramuscularly (IM) once daily (SID) for three days, intravenous fluid therapy with 500 mL of 5% glucose daily (1 mL/kg/h infusion rate over three days), a single oral dose of 100 g glycerin, dexamethasone 20 mg IM (SID for three days), and continuous supplementation with a sheep-specific mineral salt containing 30 g of cobalt sulfate per 100 kg of mineral salt. All animals exhibited complete clinical recovery with no relapse.

In Case II, among a herd of 5,000 animals, approximately 3,000 (60%) became ill, and 35 (0,7%) died within 60 days. The animals had free access to pastures dominated by *Urochloa decumbens* and *U. brizantha*, with water sourced from rivers, streams, and lakes. The animals received a commercial mineral supplement specifically for cattle, containing (per kg): 130 g phosphorus (P), 220 g calcium (Ca), 18 g magnesium (Mg), 36 g sulfur (S), 6,000 mg zinc (Zn), 1,500 mg copper (Cu), 2,000 mg manganese (Mn), 200 mg cobalt (Co), 90 mg iodine (I), and 36 mg selenium (Se). Clinical abnormalities were observed in breeding females and animals over 12 months of age (both sexes). Affected individuals displayed body condition score 2 (5/5; 100%), dehydration (5–8%) (5/5; 100%), lacrimation (3/5; 60%), pale mucous membranes (5/5; 100%), ruminal atony (4/5; 80%), anorexia with progressive emaciation (5/5; 100%), and lignophagia (5/5; 100%). Hematological analysis revealed hypochromic macrocytic anemia and leukocytosis (Table 2). Three animals (3/5; 60%) exhibited aggression, ataxia, blindness, and nystagmus, progressing to depression, opisthotonos, and death.

**Table 2:** Mean hematological values of the evaluated animals

Parameters	Observed values¹	Reference range²
Red blood cells (×10⁶/µL)	2.35 ± 0.67	5.0–10.0
Hemoglobin (g/dL)	4.22 ± 1.10	8.0–15.0
Hematocrit (%)	13.60 ± 3.20	24–46
MCV (fL)	58.32 ± 3.72	37–53
MCHC (%)	30.94 ± 1.15	33–38
White blood cells (×10³ cells/µL)	16.36 ± 5.47	4.0–12.0

¹Mean ± Standard deviation; ²Reference values from Thrall et al. (2015)

A necropsy was performed on a 17-month-old animal exhibiting neurological abnormalities that progressed to death. Antemortem clinical examination revealed a body temperature of 37.7 °C, absent pupillary reflex, nystagmus, mandibular rigidity, heart rate of 74 bpm with arrhythmia, respiratory rate of 12 movements per minute (mpm), head pressing, rigid paresis, absent superficial reflexes with intact deep reflexes, and macroscopically normal cerebrospinal fluid. Macroscopically, the brain exhibited moderate to marked diffuse edema with flattened gyri. The liver displayed signs of tension lipidosis. Histopathological analysis identified perivascular congestion and edema in the midbrain, spinal cord, cerebellar peduncles, temporal cortex, cerebellum, and occipital cortex, as well as congestion in the occipital cortex and neuropil. Liver cobalt (Co) determination via spectrophotometry revealed subdeficiency, with a mean value of 0.09 mg/kg.

The animals received 5 mg/kg of vitamin B12 intramuscularly (IM) once daily (SID) for three days and 30 g of cobalt sulfate per 100 kg of mineral salt orally. Approximately 36 h after treatment initiation, the animals resumed feeding and exhibited complete clinical recovery within 30 days. The additional cobalt supplementation via mineral salt was maintained continuously on the property. Hematological parameters returned to normal ranges within 30 days post-treatment.

In Case III, animals were raised on pastures dominated by *Urochloa brizantha*, *Panicum maximum* cv. Massai, and *Pennisetum clandestinum*, with water sourced from rivers, streams, and lakes, and no mineral supplementation. Affected individuals included males and females aged 14–60 months. Ten animals (26,32%; 4 cows and 6 heifers) exhibited anorexia, progressive emaciation over five months, dry feces, pale mucous membranes, and rough hair coats.

**Fig. 2:**
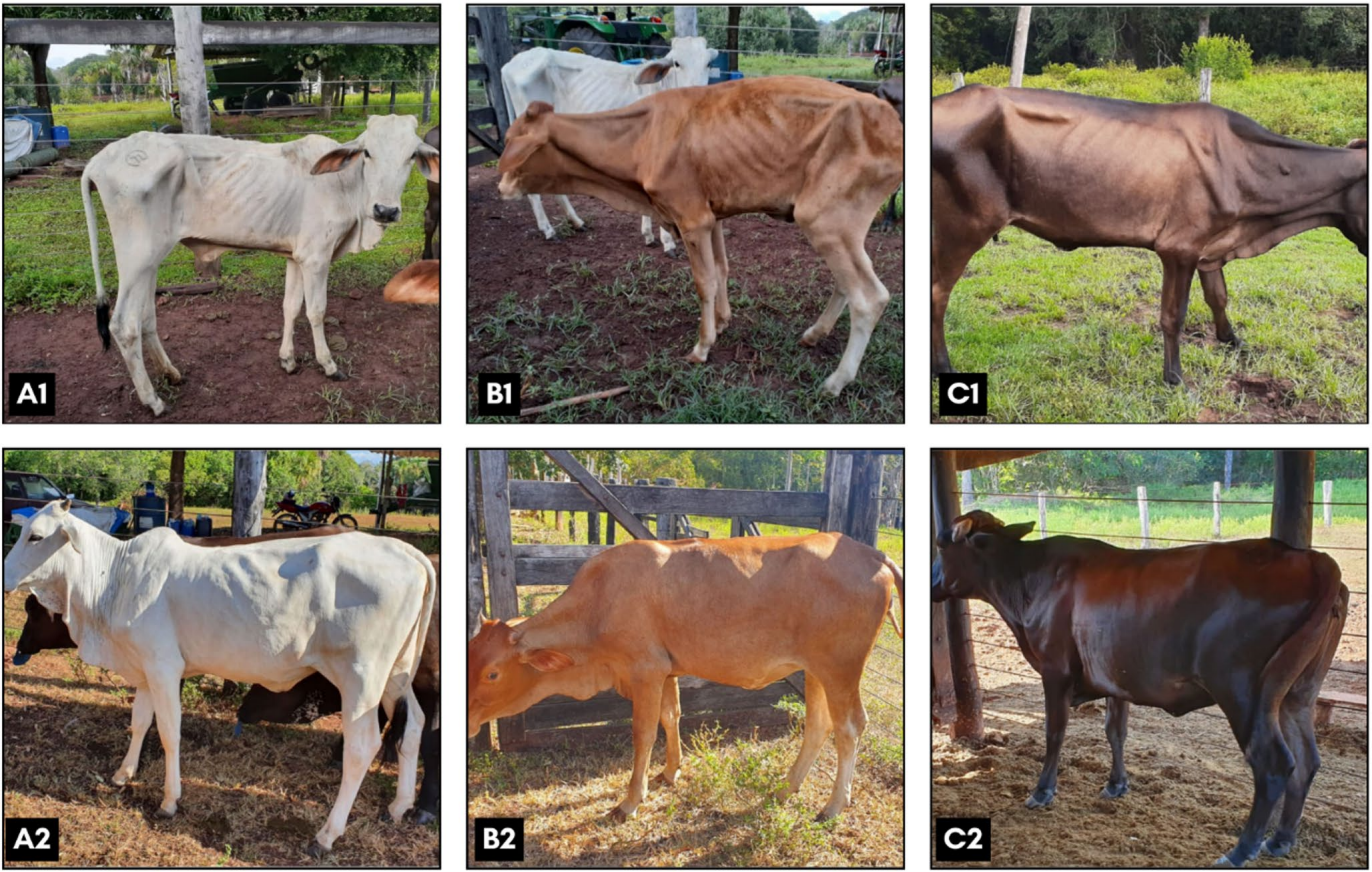
Clinical progression of cobalt-deficient cattle before and after treatment. Panels **A1**, **B1**, **C1**: Pre-treatment presentation, including cachexia, poor body condition score (BCS), rough hair coat, and hypopigmented mucous membranes. Panels **A2**, **B2**, **C2**: Post-treatment improvement in BCS, hair coat quality, and mucosal pigmentation following intramuscular vitamin B12 administration and oral cobalt supplementation. Source: Author’s archives, 2021

Clinical evaluation revealed apathy (10/10; 100%), lignophagia (8/10; 80%), cachexia (10/10; 100%) (Fig. 2), pale mucous membranes (9/10; 90%), tachycardia (130 bpm - mean) (7/10; 70%), tachypnea (80 mpm - mean) with harsh lung sounds (8/10; 80%), and ruminal atony without stratification (6/10; 60%). Erythrogram parameters improved post-treatment (Table 3), with complete clinical recovery observed within 60 days of supplementation and treatment initiation (Fig. 2).

**Table 3:** Mean hematological values of animals before and after Co supplementation

Parameters	Pre-treatment¹	Post-treatment¹	Reference range²
Red blood cells (×10⁶/µL)	5.77	7.19	5.0–10.0
Hemoglobin (g/dL)	9.10	11.73	8.0–15.0
Hematocrit (%)	24.37	31.63	24–46
MCV (fL)	42.23	44.10	37–53
MCHC (%)	37.40	37.27	33–38
White blood cells (×10³ cells/µL)	7.92	12.20	4.0–12.0
Platelets (×10³/µL)	313.00	294.33	100–800

¹Mean values; ²Reference values from Thrall et al. (2015)

## Discussion

This study represents the first descriptive report of cobalt (Co) deficiency in sheep and cattle within the state of Tocantins, Brazil. While detailed accounts of this deficiency have been historically scarce in the region, Co deficiency accounted for 15.63% of nutritional disorder cases in cattle treated at the Veterinary Hospital of the Federal University of Tocantins between 2011 and 2015 (Oliveira et al. 2019).

The three affected properties practiced extensive management systems, relying on pasture as the primary feed source. In Brazil, most forage species are adapted to low-nutrient soils (Gerdes et al. 2000); however these grasses often lack adequate crude protein (CP), total carbohydrates (TCH), and essential minerals (Nascimento et al. 2001). This nutritional inadequacy predisposes herds to deficiencies, particularly when pasture constitutes the sole dietary component. Although Property I implemented feed supplementation, this failed to correct Co deficiency, aligning with reports of insufficient mineral provision in grazing systems (Khan et al. 2023; Silva et al. 2020). Notably, one-third of properties omitted mineral supplementation entirely, a critical oversight given the global necessity of sodium chloride (NaCl) supplementation (Malafaia et al. 2014). The absence of phosphorus (P) copper (Cu), and Co—minerals frequently deficient in Brazilian soils—further exacerbated the issue (Malafaia et al. 2014; Silva et al. 2020; Ventura et al. 2021).

Common management errors included diluting mineral salt with NaCl to reduce costs and using uncovered troughs, leading to product loss and ineffective supplementation. Such practices correlate with clinical cases, mortality, and economic losses (Malafaia et al. 2014). Affected animals were primarily in production or rapid growth phases, emphasizing the need for *ad libitum* mineral access (Malafaia et al. 2014; Ventura et al. 2021; Khan et al. 2023). However, even recommended Co doses (5–10 mg/day; NRC 2016) may fail to meet requirements due to breed, age, environmental factors, and inconsistent intake (Medeiros et al. 2015).

Clinical signs—hyporexia, anorexia, ruminal atony, and progressive emaciation—reflect Co’s role in vitamin B12 synthesis and methylmalonyl-CoA enzyme activity, critical for ruminal microbiota, energy metabolism, and DNA synthesis (Suttle 2010). Co deficiency disrupts propionate metabolism, triggering appetite suppression (Tokarnia et al. 2010; Silva et al. 2020; Helmer et al. 2021). Lignophagia, though linked to fiber or protein deficits (Radostits 2007), strongly indicates Co deficiency (Suttle 2010; Ventura et al. 2021).

Anemia patterns diverged from prior reports: normocytic normochromic (Case I) and macrocytic hypochromic (Case II) anemias contrast with the macrocytic normochromic anemia described by Kaneko et al. (2008). This variability may reflect disease stage or deficiency severity, as Co-dependent cobalamin is essential for hemoglobin synthesis (Suttle 2010; Radostits 2007).

In Case I, hypoglycemia was observed with normal BHB levels. Hypoglycemia, attributed to impaired methylmalonyl-CoA function, disrupts the conversion of propionate to glucose, mobilizing fat reserves and inducing ketosis, consequently increasing BHB levels (Suttle 2010; Tokarnia et al. 2010; Radostits 2007). However, BHB levels remained normal, corroborating Morrison (2017), who found no change in these levels despite supplementation with B vitamins.

Elevated ketones (> 1.12 mmol/L) and hypoglycemia may compromise the blood-brain barrier, causing neurotoxicity and cerebral edema, as observed in Case II (Silva et al. 2020). Spongy degeneration in white matter, typically linked to hepatic encephalopathy in sheep (Constable et al. 2017), may arise from Co-deficient hypoglycemia in cattle (Barros 2016).

Analysis of cobalt in a liver sample (0.09 mg/kg; subdeficiency range: 0.05–0.12 mg/kg) confirmed diagnosis (Radostits 2007; Suttle 2010; Tokarnia et al. 2010). Analysis of materials from the animal is preferable to soil and forage analysis, as the mineral content in forage may be normal or even high, but the animal may manifest deficiency due to absorption failures due to interference from other factors (Tokarnia et al. 2010). Furthermore, the liver is the organ of choice for Co analysis because it is the main storage organ, providing a more accurate picture of the balance of elements in the body and its nutritional status (Suttle 2010; Tokarnia et al. 2010).

However, therapeutic response to Co/B12 supplementation remains the gold standard (Tokarnia et al. 2010). Here, 0.2–0.5 mg/animal of intramuscular vitamin B12 and 30 g Co/100 kg mineral salt achieved full recovery within 30 days.

Cobalt deficiency in Tocantins underscores the necessity of tailored mineral supplementation, particularly in high-demand production phases. Effective management—avoiding salt dilution, ensuring trough protection, and monitoring intake—can mitigate economic losses and animal welfare concerns. This report highlights Co deficiency in previously undocumented regions, urging proactive nutritional strategies to safeguard herd health.

## Data Availability

The datasets generated and/or analyzed during the current study are available in the Google Drive repository at [https://drive.google.com/drive/folders/1j7J56AAvqX9IB2Vj9kZmEIO64eccGPlm?usp=sharing]. The data are also available from the corresponding author upon reasonable request.
